# Cognitive Effects of Transcranial Direct Current Stimulation Plus Robotic Verticalization in Minimally Conscious State

**DOI:** 10.3390/biomedicines12102244

**Published:** 2024-10-02

**Authors:** Antonio Gangemi, Rosaria De Luca, Rosa Angela Fabio, Mirjam Bonanno, Davide Cardile, Maria Randazzo Mignacca, Carmela Rifici, Francesco Corallo, Angelo Quartarone, Federica Impellizzeri, Rocco Salvatore Calabrò

**Affiliations:** 1 IRCCS Centro Neurolesi “Bonino-Pulejo”, S.S. 113, C.da Casazza, 98124 Messina, Italy; antonio.gangemi@irccsme.it (A.G.); rosaria.deluca@irccsme.it (R.D.L.); mirjam.bonanno@irccsme.it (M.B.); davide.cardile@irccsme.it (D.C.); maria.randazzo@irccsme.it (M.R.M.); carmela.rifici@irccsme.it (C.R.); francesco.corallo@irccsme.it (F.C.); angelo.quartarone@irccsme.it (A.Q.); roccos.calabro@irccsme.it (R.S.C.); 2 Department of Cognitive, Psychological and Pedagogical Sciences and Cultural Studies, University of Messina, 98100 Messina, Italy; rafabio@unime.it

**Keywords:** transcranial direct current stimulation, tDCS, robotic verticalization training, disorders of consciousness, DoC, P300, neurorehabilitation

## Abstract

Background and Objectives: Transcranial direct current stimulation (tDCS) is a non-invasive therapeutic method that modulates cortical excitability and shows promising results for treating disorders of consciousness (DoCs). Robotic verticalization training (RVT) has been shown to enhance motor and cognitive recovery. This study evaluates the effects of an innovative approach combining RVT with tDCS in individuals with DoCs. Methods: Twenty-four subjects with DoCs, particularly those with chronic minimally conscious state (MCS) due to vascular or traumatic brain injury, participated in a quasi-randomized study at the Neurorehabilitation Unit, IRCCS Neurolesi (Messina, Italy). Participants were divided into either a control group (CG) receiving RVT alone or an experimental group (EG) receiving combined tDCS and RVT. Both groups underwent treatments five times weekly for four weeks, with tDCS/sham sessions over the dorsolateral prefrontal cortex (DLPFC) lasting 20 min before Erigo training sessions, which lasted 45 min. Results: The findings indicate that combining tDCS with Erigo^®^ Pro RTT could lead to greater improvements in cognitive functioning and P300 latency compared to the CG. Conclusions: These results suggest that the integrated approach of tDCS with RVT could offer significant benefits for patients with MCS, highlighting its potential to enhance cognitive recovery, such as reducing P300 latency.

## 1. Introduction

The term “disorder of consciousness” (DoC) refers to a medical condition resulting from a neural system injury that causes cerebral dysfunction, potentially leading to an altered state of consciousness [1]. This alteration is due to significant changes in levels of awareness and arousal and may be primary or secondary to traumatic or vascular brain injuries [2,3]. The extent of residual consciousness allows the disorder to be categorized into different conditions: minimally conscious state (MCS), unresponsive wakefulness syndrome (UWS), and coma [4,5]. In MCS, the patient is in a significantly impaired state of consciousness and shows fluctuating but replicable responses in recognizing sensorial stimuli and/or their surroundings [6]. To assess these responses, specific behaviors are typically examined, including following basic commands, maintaining visual attention or tracking, handling objects, demonstrating expressive language skills, providing verbal or gestural reactions to stimuli, and exhibiting other purposeful responses to environmental stimuli that cannot be explained solely by reflexes [7]. The recovery of specific functions or the disappearance of pathological behaviors serve as indicators of the patient’s recovery, as demonstrated by other authors. The clinical management of patients with DoCs remains challenging, and available therapeutic options are limited [8]. Despite advancements in understanding the neural substrates of consciousness, translating this knowledge into effective treatments for DoCs has proven to be a significant clinical challenge. To date, both pharmacological and non-pharmacological treatment options are available. Among non-pharmacological treatments, rehabilitation protocols are essential for improving recovery and long-term care. In the management of patients with DoCs, early mobilization and frequent postural changes are crucial to prevent complications such as contractures and pressure ulcers, and to maintain stable vital signs. Verticalization therapy is advocated early on to prevent autonomic nervous system deterioration and bedridden complications, stimulating sensory pathways and cortical involvement in trunk and lower limb control for enhanced recovery. It is generally considered a safe and well-tolerated method that can improve orthostatic tolerance, cognitive function, overall motor skills, sensorymotor abilities, and vestibular system adaptability, as observed in post-stroke and acquired brain injury patients. Nevertheless, verticalization carries the risk of inducing syncope if implemented without concurrent lower limb stepping function. In this context, original approaches like robotic devices that couple the stepping movements have proved to further promote recovery, ensuring the safety of patients [9]. In the last ten years, the use of innovative approaches (e.g., robotics, virtual reality, neuromodulation) to further promote recovery has noticeably increased [10]. Novel rehabilitative approaches allow for very early verticalization and gait training through robotic devices and other innovative tools, boosting neuroplasticity due to high-intensity, repetitive, and task-oriented training [7,11]. Robotic verticalization training (RVT) offers several advantages, including the ability to mobilize patients with severe acquired brain injury (ABI) at an early stage, even when they are in MCS or vegetative states. This early mobilization helps prevent muscle atrophy and improves circulation. Additionally, RVT enhances hemodynamic stability by reducing the risk of orthostatic hypotension and syncope through continuous passive movement. It also leads to better neurological outcomes, such as improved levels of arousal and awareness, and has been shown to be safe in the intensive care unit setting, even when initiated soon after injury. However, RVT also has some drawbacks. It is resource-intensive, requiring costly specialized equipment and trained personnel. The evidence supporting RVT is still limited, with many studies being small pilot trials; thus, more extensive research is needed. Moreover, RVT may lead to prolonged hospitalization, which can increase healthcare costs and resource utilization. Additionally, not all patients are suitable candidates for RVT, especially those with unstable intracranial pressure or severe fractures, which limits its applicability. As demonstrated in various studies, RVT could be a promising intervention that not only aids in early mobilization but also enhances both neurological function and hemodynamic stability. For example, studies collectively underscore the potential benefits of RVT in the rehabilitation of patients with ABI [9,11,12,13]. For instance, initiating RVT soon after injury could lead to significant improvements in neurological outcomes, particularly in terms of patient arousal and awareness as measured by the Coma Recovery Scale-Revised (CRSr). Moreover, robotic stepping during verticalization improves hemodynamic stability, reducing the incidence of syncope compared to the traditional verticalization method. Lastly, these studies highlighted improvements in both the Glasgow Coma Scale (GCS) and the CRSr, suggesting that RVT contributes to a more comprehensive recovery process by addressing multiple aspects of neurological and physical health. According to the literature, robotic devices such as the Erigo^®^ device [Hocoma, Volketswil, Switzerland; https://www.hocoma.com/us/media-center/media-images/erigo/ (accessed on 2 September 2024)] allow a repetitive, long-lasting, and task-oriented motor training, thus boosting neuroplastic processes. Specifically, the Erigo^®^ device combines gradual verticalization with repetitive leg movements. In previous studies, the Erigo^®^ device was found to be a valid tool in enhancing arousal recovery by boosting neuroplasticity in patients with MCS. Recent findings have reinforced the hypothesis that exercise and motor stimulation facilitated by the Erigo^®^ device can modulate brain activity. The specific changes observed in the alpha and beta bands highlight Erigo^®^’s potential as a valuable tool for promoting neural plasticity, thereby paving the way for further research and applications in neurological rehabilitation [7]. However, whether the effects of Erigo^®^ on the brain can be enhanced remains to be understood.

Another interesting non-pharmacological intervention involves the use of non-invasive neuromodulation techniques to facilitate DoC recovery by modulating brain excitability. These methods are thought to operate by reorganizing brain networks, possibly correcting the abnormal connectivity seen in various diseases, and thereby potentially alleviating associated symptoms. Various non-invasive brain stimulation (NIBS) techniques have grown exponentially in recent years. Transcranial direct current stimulation (tDCS) is a NIBS technique that has been widely used in the rehabilitation of neurological and psychiatric disorders [7,11]. This technique involves the application of a low-intensity continuous electrical current (1–2 mA) to the scalp, which modulates the resting state membrane potential polarization of neuronal populations in the underlying brain regions [12,13]. Common key brain targets for the stimulation include the dorsolateral prefrontal cortex (DLPFC), especially in working memory, attention, and executive functions, and the posterior parietal cortex, which plays a critical role in visual–spatial perception and attentional processes. Compared to other neuromodulation techniques like repetitive transcranial magnetic stimulation (r-TMS), tDCS is less likely to induce epilepsy and can induce longer-lasting therapeutic effects mediated by synaptic pathways [14]. These electric currents, whether cathodal (negative) or anodal (positive), produce an electrical field capable of changing neural functions by influencing the membrane potential of the nearby neuronal regions. They achieve this by causing either hyperpolarization (resulting in inhibition) or depolarization (resulting in facilitation). Additionally, tDCS equipment is relatively inexpensive and can be used without strict site restrictions, making it more convenient for bedside or home use in comparison to other NIBS [15,16,17]. Previous meta-analyses on the effects of non-invasive brain stimulation in DoC patients have suggested that individuals in MCS may benefit from tDCS [7,18,19,20,21,22,23,24,25]. However, the combined use of robotic verticalization therapy (RVT) with Erigo^®^ and tDCS has not been thoroughly explored. In this study, the neurophysiological and clinical effects of an innovative rehabilitative approach, combining intensive tDCS treatment with RVT using the Erigo^®^ device in chronic-phase MCS patients, were demonstrated. The findings indicate that this novel intervention significantly enhanced patient outcomes by boosting both neuroplasticity and hemodynamic stability. Specifically, improvements were observed in measures such as the Coma Recovery Scale-Revised (CRS-R), Glasgow Coma Scale (GCS), and overall arousal and awareness levels, suggesting that this approach can be an effective therapeutic option for patients with severe disorders of consciousness.

## 2. Materials and Methods

A total of twenty-four subjects (fifteen males and nine females) diagnosed with chronic MCS due to vascular or traumatic brain injury (at least 12 months after the event) attending the Intensive Neurorehabilitation Unit of the IRCCS Neurolesi Center Bonino Pulejo (Messina, Italy) from February 2022 to May 2023, were enrolled in this prospective case-control study. A more detailed description of the MCS patients’ demographic condition is reported in Table 1. All experiments were conducted according to the ethical policies and procedures approved by the local ethics committee (IRCCS-ME CEL/U21/22 16 December 2022).

The inclusion criteria were: (i) age > 18 years; (ii) diagnosis of chronic MCS (i.e., at least 6 months after the traumatic or vascular event) according to the CRSr [26,27] which is administered at enrollment; (iii) adequate pulmonary gas exchanging function (arterial O_2_ pressure/O_2_ flux ratio 250); (iv) stable hemodynamics (absence of dangerous variations in mean arterial pressure or heart rate) even if obtained with continuative amines support. The exclusion criteria were: (i) sedation; (ii) unstable intracranial pressure; (iii) cerebral perfusion pressure < 60 mmHg; (iv) fractures or skin lesions in thorax, abdomen, or lower limbs; (v) deep vein thrombosis; (vi) other medical conditions potentially interfering with verticalization; (vii) body weight > 130 kg and height > 210 cm.

### 2.1. Procedures

The MCS patients included in the study were divided into two groups with identical demographic and medical characteristics. Participants were assigned to one of the two groups using a quasi-randomized approach. The assignment was based on the enrollment order. In this prospective case-control study, the experimental group (EG) received RVT combined with tDCS, referred to as RVT-Plus. The control group (CG) received the RVT with sham-tDCS, referred to as RVT alone. tDCS (lasting 20 min) was applied to both groups (active e or sham) before the Erigo^®^ training. Both groups received RVT in a dedicated space, five times a week for four weeks (i.e., a total of twenty sessions), with each Erigo^®^ training session lasting 45 min. Each patient underwent evaluation using a tailored multidimensional screening, applying clinical scales at both the start (T0) and conclusion (T1) of the study. The assessments were conducted by a clinician who was not directly involved in the study. Furthermore, a neurophysiology technician recorded brain electrical activity through event-related potential P300.

### 2.2. Clinical Outcomes

All subjects were evaluated before (T0) and after (T1) the rehabilitation training. Specifically, a neuropsychologist administered the levels of cognitive functioning (LCF) scale [28], which is used to assess cognitive performance in post-coma patients, while a physiotherapist administered the functional independence measure (FIM), an eighteen-item tool [thirteen motor (motFIM) and five cognitive (cognFIM)] designed to explore social, psychological, and physical functioning, assessing the patient’s level of dependence in daily life activities [29]. Additionally, neurologists evaluated all patients before training using the Simplified Evaluation of CONsciousness Disorders (SECONDs) clinical scale, which helped determine the level of consciousness in enrolled MCS patients. The test covers items such as observation, command following, communication (intentional or functional–conditional item), visual pursuit, visual fixation, localization to pain (conditional item), oriented behaviors, and arousal [30].

### 2.3. Neurophysiological Outcomes

The electroencephalographic signal was recorded with a sampling frequency of 500 Hz, applying a bandpass filter ranging from 0.1 Hz to 70 Hz. The SCAN software (version 4.3, Neuroscan, Compumedics, El Paso, TX, USA) along with NuAMP amplifiers were used. A total of nineteen (Ag/AgCl) scalp electrodes were applied according to the international standard 10/20 measurement system described by Jasper [31]. The signal was recorded using a monopolar montage; therefore, the active electrodes positioned at the vertex FZ-CZ-PZ were referenced to a common electrode placed on the mastoids in position A1. Electrode impedances were maintained between 5 and 10 kΩ. Electroencephalogram (EEG) signals intended for event-related potential (ERP) analysis were processed offline, applying a low-pass filter at 20 Hz, baseline correction, and segmentation of waveforms into epochs centered on the stimulus presentation. Trials with amplitudes beyond ±100 μV were eliminated. A minimum of 20 trials per stimulus were considered necessary for the inclusion of individual ERP waveforms. The EEG epochs ranged from 200 ms to 1000 ms, generated offline and centered on low and high tones. The ERP P300 potentials were automatically detected within specific time intervals (70–110 ms, 210–270 ms, and 270–370 ms) from midline positions (Fz, Cz, and Pz), where these peaks show maximum activity. Frequent stimuli immediately preceding each rare stimulus were selected for averaging, ensuring comparable signal-to-noise ratios. For the detection of the ERP P300, the auditory Oddball paradigm has been used. It involves the administration of auditory stimuli without the need for direct responses. The stimuli were delivered using specialized Presentation® software (version 24.1, build 17 June 2024) from Neurobehavioral Systems Inc, Berkeley, CA, USA, [https://www.neurobs.com/ (accessed on 27 September 2024)] in Neurowave system (version 2.22.0.0) of Khymeia [https://khymeia.com/it/products/neurowave/ (accessed on 27 September 2024)]. The auditory paradigm included two categories of sounds: frequent pure sinusoidal tones and rare tones. Specifically, 20% of the stimuli were rare tones (2 kHz), while 80% were frequent tones (1.5 kHz), both with a duration of 200 ms, a rise and fall time of 5 ms, and a sound pressure level of 70 dB SPL. Each tone or noise lasted 200 milliseconds, with an interstimulus interval of 700 ms. The presentation was divided into two blocks, each consisting of 700 stimuli (560 frequent, 140 rare). Participants actively engaged in listening for the rare stimulus. The total time required to complete the task was approximately 20 min.

### 2.4. Robotic Verticalization Training (RVT)

**Figure 1:**
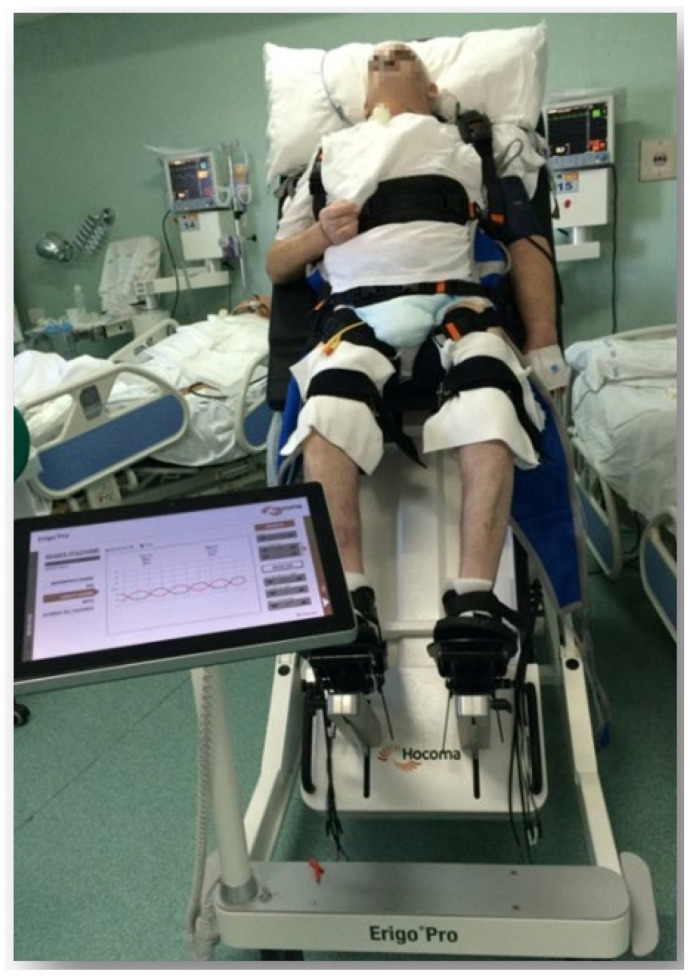
The image shows a RVT session in a patient with MCS using the Erigo^®^ device. The patient is positioned on the device, which provides controlled verticalization and passive mobilization of the lower limbs, facilitating both motor stimulation and safe upright positioning.

The Erigo^®^ device is a robotic tilt table that allows a single therapist to safely and efficiently provide mobilization, verticalization, and sensorimotor stimulation simultaneously. It combines gradual verticalization with robotic leg movement therapy (Figure 1). Thanks to the unique afferent stimulation provided by this device and its flexible harness, patients can undergo intensive and safe training even in the early stages of rehabilitation. The device’s robotic leg movements and cyclic leg loading are essential afferent stimuli for the central nervous system, leading to muscle activation, improved muscle pump function, and enhanced venous return (which improves cardiovascular stability). Patients tolerate the upright position better with the Erigo^®^ than with conventional tilt tables that lack a stepping function and cyclic leg loading. After an initial session of less than 25 min to adapt the patient to the device, each subsequent robotic session lasted about 45 min. During the twenty sessions of training, the table inclination gradually increased from 45 to 90 degrees, and the stepping velocity was adjusted according to the patient’s needs and clinical conditions. In the CG, patients received only RVT with the Erigo^®^ device, without tDCS, to preserve the effects of the device itself. The CG underwent RVT five times a week, with each session lasting 45 min, over a period of four weeks, totaling twenty sessions, in addition to standard neurorehabilitation.

### 2.5. RVT-Plus: Combined Robotic-tDCS Approach

Before starting the treatment, participants underwent a preliminary neurophysiological evaluation using the Evoked Potential ERP P300 and a clinical assessment. The EG underwent tDCS on the DLPFC, while the CG received sham-tDCS. During and after each tDCS session, participants were continuously monitored for any adverse effects, and any symptoms were promptly recorded and managed. At the end of the treatment, all participants were reassessed using event-related potential (ERP) P300 to evaluate any neurophysiological changes, as well as the clinical scales used in the pre-test phase. The assessors conducting the evaluations were blinded to the tDCS treatment. For this research, the BrainSTIM transcranial electrical stimulator, produced by EMS S.r.l. in Bologna, Italy, was used [http://www.emsmedical.net/prodotti/tdcs/942-brainstim (accessed on 2 September 2024)]. Stimulation was administered using two sponge electrodes, each 25 mm in diameter, pre-coated with saline gel. A battery-powered stimulator delivered a constant current. To stimulate the left dorsolateral prefrontal cortex (DLPFC), the anode was placed over the F7 region, while the cathodic reference electrode was positioned in the right supraorbital region, with precise electrode localization based on the EEG 10-20 system. The stimulation intensity was set at 2 mA (with a current density of 2.5 mA/cm^2^) for 20 min.

### 2.6. Statistical Analysis

Data analysis was conducted using IBM SPSS Statistics, Version 24 (IBM, Armonk, NY, USA). Multivariate Analysis of Variance (MANOVA) models for repeated measures were employed. The analysis included a between-subject factor (Group: experimental—Erigo^®^ Pro RTT with tDCS—and control—Erigo^®^ Pro RTT alone) and a within-subject factor (phases: T0—pre-intervention baseline; T1—post-test) for each parameter. Neurophysiological measures focused on P300 latency, while clinical measures encompassed LCF and FIM.

To address multiple comparisons, a Bonferroni correction was applied. The significance level was set at *p* < 0.05 for all statistical tests. In cases of significant effects, the effect size was reported using eta squared (η^2^) and categorized accordingly.

## 3. Results

Baseline measurements taken prior to intervention (T0) were compared with post-test results (T1). Table 2 shows means and standard deviations of P300 latency, LCF, FIM scores, *p*-values, and *t* test for independent samples.

**Figure 2:**
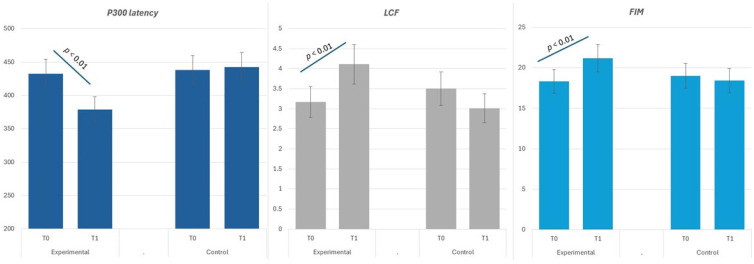
Change scores in P300 latency, LCF, and FIM between baseline (T0) and end of treatment (T1). Note. The figure shows changes in P300 latency (blue bars), level of consciousness (LCF; gray bars), and functional independence measure (FIM; light blue bars) between baseline (T0) and post-treatment (T1) for the experimental and control groups. Error bars represent standard deviations. Statistically significant improvements (*p* < 0.01) were observed in the experimental group across all measures, while the control group showed no significant changes. P300 latency was assessed using neurophysiological measures, while LCF and FIM scores were evaluated using clinical assessment tools. Paired *t*-tests were performed to analyze within-group differences from T0 to T1.

Considering the P300 latency, the ANOVA model for repeated measures revealed a significant effect of the factor Phase (F (1, 22) = 19.11, *p* < 0.01, η^2^ = 0.09), indicating a difference between the pre- and post-test results (Figure 1). The Group by Phase interaction also showed significant effects (F (1, 22) = 13.11, *p* < 0.01, η^2^ = 0.11). These results indicated that there was a significant reduction in P300 latency from T0 to T1 in the EG, indicating improved neurophysiological processing speed (*p* = 0.001). In contrast, the CG did not show a significant change in P300 latency over the same period (*p* = 0.45). With reference to the LCF, a significant effect of the factor Phase emerged again (1, 22) (F = 19.37, *p* < 0.01, η^2^ = 0.09), indicating a difference between the pre- and post-test results (Figure 2). The Group by Phase interaction showed no significant effects indicating that both groups benefited from the trainings (F (1, 22) = 1.67, *p* < 0.21, η^2^ = 0.11). Additionally, the t test for independent samples indicated that while there was no significant difference in LCF scores between EG and CG at T0 (*p* = 0.36), the EG demonstrated a significant improvement in LCF scores from T0 to T1 (*p* = 0.05), suggesting enhanced cognitive functioning post-intervention. Conversely, the CG showed no significant change in LCF scores over the same period. With reference to FIM, a significant effect of the factor Phase emerged (F (1, 22) = 24.49, *p* < 0.01, η^2^ = 0.09), indicating a difference between the pre- and post-test results. The Group by Phase interaction also showed significant effects (F (1, 22) = 17.35, *p* < 0.01, η^2^ = 0.11). These results indicate that at T0, there was no significant difference in FIM scores between EG and CG (*p* = 0.49). However, post-intervention at T1, the EG exhibited a significant increase in FIM cognitive scores compared to T0 (*p* = 0.01), indicating enhanced cognitive functioning following treatment. In contrast, the CG showed a non-significant change in FIM scores from T0 to T1.

## 4. Discussion

The key contribution of this study is that it is the first to investigate the effects of a novel synergistic rehabilitative approach, combining tDCS with RVT using the Erigo^®^ device in patients with chronic MCS, thus exploring a new area of research in the current field of neurorehabilitation. The EG showed significant neurophysiological and clinical improvements in both LCF and FIM post-intervention scores (mainly related to cognitive scores), indicating enhanced cognitive functioning compared to the CG. These findings underscore the potential synergistic effects of combining tDCS with RVT with Erigo^®^ Pro in promoting recovery in MCS patients. The absence of significant changes in the CG highlights that the observed improvements were specific to the intervention received by the EG. These findings emphasize the importance of tailored interventions targeting specific neurophysiological mechanisms underlying consciousness disorders. In previous studies, authors demonstrated that the Erigo^®^ device induced changes in alpha and beta bands post-intervention, highlighting its promising effect on influencing neural plasticity in MCS patients. The neural mechanisms underlying DoCs are intricate and still not fully understood [32,33]. The brain regulates arousal in response to specific stimuli and maintains awareness through the activity of two key neuronal circuits: the Ascending Reticular Activating System (ARAS) and the thalamocortical loops. The ARAS, which links the upper brainstem tegmentum (reticular formation) to the thalamus, hypothalamus, and basal forebrain, facilitates widespread cortical activation, thus serving as the neural substrate for arousal and conscious awareness [34,35]. Lesions affecting ARAS can lead to coma, the most severe form of DoC. Notably, glutamatergic and cholinergic neurons in the dorsal tegmentum of the midbrain and pons are considered central to maintaining wakefulness. These regions activate the central thalamus, particularly the intralaminar nuclei and the basal forebrain, which subsequently stimulate the cortex via glutamatergic and cholinergic projections, respectively [36]. The mesocircuit fronto-parietal model proposes that regions such as the frontal cortex, central thalamus, brain stem, striatum, and globus pallidus interna play crucial roles in consciousness processing, and these areas are also targets for interventions aimed at treating DoCs [37]. Moreover, in the last decade, most studies investigated the use of non-invasive neuromodulation techniques integrating conventional rehabilitation treatment of MCS patients with interesting considerations [4,22,38,39,40,41,42,43,44,45,46,47]. The results of this study support the hypotheses regarding the potential effects of tDCS applied to DLPFC, which could potentiate the circuits underlying consciousness.

The beneficial multidimensional effects (clinical, psychometric, and neurophysiological) of combining tDCS with Erigo^®^ Pro RTT in patients with MCS further confirm the importance of a strategic multimodal approach both in the assessment of these patients and in advanced therapeutic methods, as suggested by the current literature [48,49,50,51,52,53,54,55]. In fact, the significant reduction in P300 latency in the EG suggests improved neurophysiological processing speed, indicative of enhanced cognitive processing and information transfer. This finding aligns with previous research indicating the efficacy of tDCS in modulating cortical excitability and enhancing cognitive function [56,57,58,59,60]. Overall, the results suggest that the integration of tDCS with Erigo^®^ Pro RTT holds promise as a therapeutic approach for enhancing cognitive function in patients with DoCs. This improvement in neural plasticity with a subsequent potentiation in psychometric measures, may explain why EG subjects achieved better results after this new advanced robotic-tDCS training rather than following the standard robotic neurorehabilitation.

## 5. Limitations and Future Directions

The relatively low number of patients included in this study might influence the results. For this reason, future randomized control trials (RCTs) are needed to further validate these findings, with larger sample sizes and longer follow-up periods to better elucidate the long-term effects and mechanisms underlying these interventions. Moreover, future research should consider conducting RCTs with extended follow-up periods to further validate their findings. Evaluating the long-term effects and underlying mechanisms of the combined tDCS and RVT on cognitive recovery and P300 latency in patients with chronic MCS is crucial for a more comprehensive understanding of the treatment’s efficacy. Furthermore, further studies could combine neuroimaging and electrophysiological techniques to investigate the specific neurophysiological mechanisms of this combined approach. This methodology would provide a more detailed understanding of how these interventions promote cognitive recovery and reduce P300 latency in patients with MCS.

## 6. Conclusions

This study demonstrates that combining tDCS with RVT could offer significant multi-modal advantages, improving cognitive and neurophysiological outcomes, compared to RVT alone in patients with chronic MCS. The results showed a significant reduction in P300 latency from T0 to T1 in the experimental group (EG), indicating improved neurophysiological processing speed (*p* = 0.001); however, this was only after the combined approach of tDCS and RVT, using the Erigo^®^ device. Additionally, the EG exhibited significant clinical improvements in post-intervention FIM scores, particularly related to cognitive function, compared to the control group (CG).

These findings suggest that implementing this promising combined approach in clinical practice could promote greater improvements in cognitive functioning, reduce P300 latency, and increase neurophysiological processing speed. Neuromodulation applied before robotic training may effectively enhance neural plasticity and cognitive recovery in individuals with MCS, highlighting the potential of this innovative therapeutic strategy to provide substantial neurorehabilitative benefits.

## Figures and Tables

**Table 1:** Socio-demographic clinical description of the population’s study and clinical scores obtained in the clinical scale called SECONDs (Simplified Evaluation of CONsciousness Disorders) scale.

Patients’	EG (*n* = 12)	CG (*n* = 12)	*p*-Value
**Age**	59.66 ± 8.33	60.08 ± 11.06	0.8
**Educational level** - Elementary school - Middle school - High school	2 (16.67%) 8 (66.67%) 2 (16.67%)	3 (25.00%) 6 (50.00%) 3 (25.00%)	0.12
**Gender** - Male - Female	7 (58.33%) 5 (41.66%)	8 (66.67%) 4 (33.33%)	0.72
**Etiology** - Vascular - Traumatic	8 (66.67%) 4 (33.33%)	8 (66.67%) 4 (33.33%)	1.00
**SECONDs**	3.08 ± 1.43	3.50 ± 1.76	0.34
**MCS+ MCS−**	1 (8.33%) 11 (91.66%)	2 (16.67%) 10 (83.33%)	0.78

Note. MCS+ pertains to patients exhibiting specific pivotal behaviors, including but not limited to consistent and reproducible responses to commands, recognition of objects, and intelligible verbalization with deliberate (albeit non-functional) communication. Conversely, MCS− denotes individuals characterized by manifestations such as reaching, visual pursuit, fixation, object manipulation, and automatic motor responses. Legend: EG (Experimental group), CG (Control group), SECONDs (Simplified Evaluation of CONsciousness Disorders), and MCS (minimally conscious state).

**Table 2:** Pre-test and post-test means and standard deviations of P300 latency, level of consciousness (LCF), functional independence measure (FIM), and *p*-values.

Measures	T0—Pre-Test		*p*	T1—Post-Test		*p*
	**Experimental**	**Control**		**Experimental**	**Control**	
*P300 latency*	432.67 (86.24)	442.34 (36.75)	0.45	379.01 (65.32)	437.98 (37.22)	0.001 ***
*LCF*	3.17 (0.83)	3.01 (1.12)	0.36	4.11 (0.90)	3.50 (1.56)	0.05 *
*FIM*	18.33 (3.01)	18.42 (1.06)	0.49	21.19 (3.66)	19.00 (1.18)	0.01 **

Note. T0 = pre-test; T1 = post-test; LCF = level of consciousness; FIM = functional independence measure. * = *p* < 0.05; ** = *p* < 0.01; *** = *p* < 0.001. Significant differences were observed between pre-test and post-test scores for the experimental group in both P300 latency and FIM. LCF showed marginal significance at *p* = 0.05. The control group did not exhibit significant changes in P300 latency, LCF, or FIM across testing periods.

## Data Availability

Data will be available on request to the corresponding author.
